# The Royal Hospital for Incurables

**Published:** 1893-12-02

**Authors:** 


### The Hospital

An Institutional Journal of

Science, Medicine, Hygiene, Nursing, and News.

*For Hospitals, Asylums, Infirmaries, Dispensaries, Medical Practitioners, Students, and Nurses.*

Vol. XV.—No. 375.] [Dec. 2, 1893.

### THE ROYAL HOSPITAL FOR INCURABLES.

Writing in February last on the possible results of a Committee then appointed under the presidency of Lord Aberdeen, we ventured to predict that in all probability better things would obtain before very long at the Royal Hospital for Incurables, Putney. We added, however, the warning, that the time had passed when great endowments, or a large revenue from any source, can be permitted to exempt any public institution from conforming to modern requirements in regard to management and efficiency. The House of Lords' Committee expressed the view that the authorities of this hospital appeared to be incapable of effecting reforms, and even extremely resentful of external observation. Since that report was issued we have personally inspected the institution at Putney, and have so been forced to the conclusion, from personal observation and inquiry on the spot, that the Lords had good reason for the judgment they delivered, and that there is no hospital in the world more dominated by its officials than the Royal Hospital for Incurables.

In another column we report the proceedings at the thirty ninth annual meeting, to which the report of the Special Committee of the institution on the Lords' suggestions was presented without notice. The report of this Committee of Inquiry was unanimous with the exception of Lords Aberdeen and Arran, who did not agree with some of the conclusions of their colleagues. This Committee, in the absence of Lord Aberdeen, as our knowledge of the inner working of the institution would have led us to suppose, found in effect that the present system was perfect, and that no reforms or alterations of any kind or description were necessary or even desirable. In other words, the report first sets out the five specific reforms recommended by the Lords' Committee, and then gives the reasons why in the signatories' opinion no alterations are necessary in any of these directions. Here then we have an institution worked upon certain lines for nearly forty years, under a system which a majority of the Committee declare does not need modification of any kind.

*Prima facie*, such a conclusion would appear to be absurd on the face of it, but we will now examine the facts on their merits. First of all, an idea may be gained of the present system of management from the circumstance that no notice was given to the Governors that the report of the Committee of Inquiry on the Lords' recommendations would be presented at the annual meeting. This meeting was summoned by public advertisement, which merely stated that the annual report and financial statement would be presented and the usual business transacted. Again, the report does not analyse the evidence, nor is the evidence published with it, although a shorthand writer was present during the sittings of the committee, who took a full report. This report appears to have been settled so long ago as July last, when the President (Lord Aberdeen) was in England, and might have been present had a special meeting of the Governors been then called to consider it. This fact and the subsequent resignation of the office of President by Lord Aberdeen, are very significant.

Lord Aberdeen, as we have reason to believe, not only dissents from the counsels-of-perfection attitude taken up by the majority of the Committee, but is of opinion that certain radical reforms are essential to the well-being and efficient management of the Royal Hospital for Incurables. This much might be gathered from the statement that he declined to sign the report of the Committee, preferring, like Lord Arran, to state his own views in a separate memorandom. The Lords' Committee recommended that a resident medical officer should be appointed, that a ladies' committee should be formed, that all the nurses should be hospital trained, that all contracts for food and stores of all kinds should be by open tender, and that the general supervision by the Committee of Governors should be greatly increased. Surely such reforms as these ought to have been welcomed by a committee of intelligent men of business who desired to promote the progressive development, and so to increase the practical usefulness of a great institution. We can say from personal knowledge, and here we are in practical agreement with the late President (Lord Aberdeen), that the adoption of several of these suggestions, is imperatively called for in the best interests of the patients, who, being incurables, should be safeguarded from the possibility of tyranny and neglect, to which they might otherwise be subjected. At

Putney we found evidence of the necessity for a radical change in the nursing arrangements, for a reorganization of the Committee and Secretariat, and for the appointment of a resident medical superintendent without a moment's delay. The report of the Committee of Inquiry, which was not satisfactory to the President of the institution, who declined to sign it, must therefore be regarded as disastrous, because it will necessitate independent action on the part of the great body of Governors who are not on the committee of the Institution.

No doubt there is an increasing demand for accommodation for incurable patients in this country; but such provision must be zealously safeguarded, or this class of patients may be liable to greater evils from confinement in a quasi-public institution where the arrangements are practically left in the hands of a few officials, than if they continued to reside with their friends though in humble circumstances, or were even inmates of workhouses. Adequate safeguards, as we have shown, are largely absent from the Royal Hospital for Incurables, and we are of opinion that the new Treasurer must either insist upon the report of the Committee of Inquiry being submitted to a special meeting of the Governors, who should—in our view—appoint a strong committee to deal with this report and the suggestions of the Lords' Committee, or failing this, independent Governors will have no alternative but to withhold further subscriptions and contributions until the present Committee is brought to live up to modern requirements. The interests of the public and the patients demand the publication of the evidence given before the Committee of Inquiry in its entirety. This step is imperatively called for, as the Committee state in their report "That if the evidence laid before them had been laid before the Lords' Committee, and they had had the advantages which we have had of a personal examination of the institution and its staff, they would have arrived at very different conclusions." We, on the other hand, having full knowledge of some of the evidence given, and having made a personal inspection of the institution and its staff, know that the Lords' Committee were undoubtedly justified in most of the conclusions they arrived at, and that unless their very reasonable recommendations be carried out, this charity cannot be regarded with public confidence, or hope to receive large contributions from the charitable. We hold that the production of the evidence in its entirety would justify and emphasise the further finding of the Lords' Committee to the effect "that the objects of this charity are excellent; but until the management is thoroughly reformed the institution is not one to be commended."

Under these circumstances it is no wonder that the financial statement showed a falling off for the year of over £5,000. This falling off in income is likely to be further increased unless steps are speedily taken by the Governors at large to restore public confidence by insisting upon the publication of the whole of the evidence, and the immediate summons of a special meeting of Governors to take the whole matter into full consideration. If there be any individual Governor who doubts the wisdom of the course here suggested, we would refer him to the speech made by Mr. Kimber, M.P., to show how completely the Committee have failed to grasp the opinions of some, at any rate, of the witnesses who were called before them. Mr. Kimber quoted the authority of Mr. Burdett, one of the witnesses, to the effect that the present management was everything that could be desired, and that to interfere with it at present would be most dangerous. Having carefully considered the evidence given by Mr. Burdett, we are in a position to state that so far from its pointing in the direction indicated by Mr. Kimber, it emphasises the importance of the prompt adoption of several of the suggestions made by the Lords' Committee.

Mr. Kimber described the treatment of the Lords' Committee as very cruel, because it might tend to diminish the number of patients admitted owing to the great drop in contributions from the public. We, on the other hand, hold that if due regard be had to the well-being and welfare of these helpless and isolated patients, it is far better that the numbers treated should gradually fall to zero, than that they should be admitted to an institution the management of which leads impartial and experienced judges to feel a doubt as to the character of the treatment they will be subjected to as inmates. There are many kind persons in the world who are quite incapable of caring properly for sick people. This is no doubt their misfortune, not their fault. So in the case of the Royal Hospital for Incurables, it is more than probable that the present managers have become so impressed with their own good intentions that a system of administration, long obsolete and inadequate, is regarded by them not only as perfect, but as even above criticism. Modern medicine proves to demonstration that the incurables, who represent the failures of the physician's and surgeon's art, still require the most careful and continuous medical treatment, unless the blessed name of hospital is to be degraded by being used as a cloak to something more serious than indifference to avoidable suffering or ordinary neglect. Such medical treatment cannot be afforded by a non-resident practitioner, and so it is nothing short of cruelty to refuse to appoint a resident medical superintendent. Hence, the Governors of the Royal Hospital for Incurables have a public duty to discharge, and we call upon them to act with the promptness and dispatch which the best interest of the patients, for whose well-being they are responsible, so imperatively demands. Let a special meeting of Governors therefore be summoned without further delay.

